# Focused Ultrasound-enabled Brain Tumor Liquid Biopsy

**DOI:** 10.1038/s41598-018-24516-7

**Published:** 2018-04-26

**Authors:** Lifei Zhu, Galen Cheng, Dezhuang Ye, Arash Nazeri, Yimei Yue, Weijun Liu, Xiaowei Wang, Gavin P. Dunn, Allegra A. Petti, Eric C. Leuthardt, Hong Chen

**Affiliations:** 1 Department of Biomedical Engineering, Washington University in St. Louis, Saint Louis, MO 63130 USA; 2 Department of Mechanical Engineering and Materials Science, Washington University in St. Louis, Saint Louis, MO 63130 USA; 3 Mallinckrodt Institute of Radiology, Washington University School of Medicine, Saint Louis, MO 63110 USA; 4 Department of Radiation Oncology, Washington University School of Medicine, Saint Louis, MO 63108 USA; 5 Department of Neurosurgery, Washington University School of Medicine, Saint Louis, MO 63110 USA; 6 Center for Human Immunology and Immunotherapy Programs, Washington University School of Medicine, Saint Louis, MO 63110 USA; 7 McDonnel Genome Institute, Washington University School of Medicine, Saint Louis, MO 63110 USA; 8 Department of Medicine, Washington University School of Medicine, Saint Louis, MO 63110 USA; 9 Department of Neuroscience, Washington University School of Medicine, Saint. Louis, MO 63110 USA; 10 Center for Innovation in Neuroscience and Technology, Washington University School of Medicine, Saint Louis, MO 63110 USA

## Abstract

Although blood-based liquid biopsies have emerged as a promising non-invasive method to detect biomarkers in various cancers, limited progress has been made for brain tumors. One major obstacle is the blood-brain barrier (BBB), which hinders efficient passage of tumor biomarkers into the peripheral circulation. The objective of this study was to determine whether FUS in combination with microbubbles can enhance the release of biomarkers from the brain tumor to the blood circulation. Two glioblastoma tumor models (U87 and GL261), developed by intracranial injection of respective enhanced green fluorescent protein (eGFP)-transduced glioblastoma cells, were treated by FUS in the presence of systemically injected microbubbles. Effect of FUS on plasma eGFP mRNA levels was determined using quantitative polymerase chain reaction. eGFP mRNA were only detectable in the FUS-treated U87 mice and undetectable in the untreated U87 mice (maximum cycle number set to 40). This finding was replicated in GL261 mice across three different acoustic pressures. The circulating levels of eGFP mRNA were 1,500–4,800 fold higher in the FUS-treated GL261 mice than that of the untreated mice for the three acoustic pressures. This study demonstrated the feasibility of FUS-enabled brain tumor liquid biopsies in two different murine glioma models across different acoustic pressures.

## Introduction

Mutations in the DNA, changes in epigenomic makeup, and variations in gene expression associated with brain tumors can inform clinical practice by providing invaluable information for diagnosis, prognostication, disease monitoring, and development of personalized treatment strategies^1^. Such molecular biomarkers, which can be examined in surgical resection or biopsy specimens, are becoming an integral component of clinical practice^2^. However, direct surgical tissue biopsy to determine tumor molecular profiles is associated with potential complications such as hemorrhage and infection^3^. Furthermore, repeated tissue biopsies using surgical interventions to assess treatment response and recurrence may not be feasible given the increased risk for complications and morbidity, especially for brain tumors. Liquid biopsy offers a noninvasive approach by detecting circulating molecular biomarkers.

Although liquid biopsy has been established in clinical care of patients with various cancer types^4^, limited progress has been made for brain tumors^2^. For brain tumor liquid biopsy, the major challenge is the hindrance of tumor biomarker release into the bloodstream by the blood-brain barrier (BBB)^5^. Even though the BBB is partially disrupted in the core part of glioblastoma (the most common type of high-grade glioma), it remains intact in large parts of glioblastoma and lower grade diffuse gliomas, which may prevent the efficient passage of biomarkers into the blood circulation. In line with this, circulating tumor DNA (ctDNA) was reported to be detectable in a small fraction of patients with advanced gliomas (<10%) as compared to patients with other solid tumors^6^. It has also been shown that patients with high-grade glioma have a significantly higher plasma DNA concentration than patients with low-grade gliomas. These suggest that the increased permeability of BBB associated with the progression of gliomas is correlated with the release of biomarkers from the tumor to the blood^7^. It was also found that although D-2-hydroxyglutarate (D2HG) levels have been used in the clinic for the diagnosis and monitoring of patients with IDH1/2-mutant malignancies, D2HG plasma levels in patients with IDH2/2-mutant gliomas are within the normal range, suggesting that the BBB prevents D2HG from entering the blood circulation^8^. Therefore, approaches that can non-invasively enhance the release of biomarkers from the brain tumors to the blood circulation would be of significant clinical relevance.

Focused ultrasound (FUS) offers great potential for achieving noninvasive and spatially-localized biomarker release into the blood stream^3,9^. The initial concept for blood biomarker amplification and localization using ultrasound was proposed in a study published in 2009, which demonstrated the feasibility to enhance the release of protein biomarkers from a colon cancer cell line in both *ex vivo* cell cultures and an *in vivo* mouse tumor model^10^. Building on this initial work, *in vitro* cell culture studies showed the feasibility of enhancing mRNA biomarker release from a breast cancer cell line using microbubble-enhanced ultrasound^11^, and the feasibility of releasing a combination of ovarian cancer biomarkers using ultrasound^12^. Two recent *in vivo* studies further demonstrated that ultrasound-mediated release of biomarkers into the bloodstream is a promising approach for detecting tumor biomarkers via blood sampling^13,14^. In one of the studies, pulsed HIFU with high acoustic pressures was used to induce histotripsy (*i.e.*, a technique for mechanical tissue fractionation) in a rat model of prostate cancer, and this enhanced release of cell-free tumor microRNA into the blood circulation^13^. In the other study, a chicken embryo tumor model was used to show the feasibility of amplifying the release of extracellular vesicles using high-intensity focused ultrasound (HIFU) in combination with phase-change nanodroplets which changed to microbubbles upon HIFU sonication^14^. Although promising, these preliminary findings cannot be readily extended to applications in the brain, given challenges inherent to the brain: first, delivery of acoustic energy to the brain is impeded by attenuation and distortion of acoustic waves by the skull; second, biomarker release from the brain is inherently limited by the presence of the BBB.

FUS in combination with microbubbles has been studied extensively for inducing BBB opening for noninvasive and localized delivery of drugs in the blood circulation to the brain parenchyma^15–17^. Many studies have been performed to optimize the treatment parameters^18–20^ and evaluate the short-term and long-term safety profiles^21–24^. Ongoing clinical trials are evaluating the feasibility and safety of FUS-induced BBB opening in patients with glioblastoma and Alzheimer’s disease^25,26^. Although all previous studies focused on exploring the utility of FUS-induced BBB as a means of targeted delivery of circulating therapeutics, we hypothesized that FUS-mediated BBB disruption could be viewed as a tool for enhancing “two-way trafficking” between brain and blood. While circulating molecules can be allowed to enter the brain using FUS-mediated BBB disruption, brain biomarkers (*e*.*g*., tumor markers) can also be released into the blood circulation for liquid biopsies. Building on the seminal works demonstrating successful drug delivery to the brain using FUS-induced BBB disruption, we propose to develop FUS-enabled brain tumor liquid biopsy technique, which uses FUS in combination with microbubbles to enhance the release of biomarkers from brain tumors into the blood circulation for liquid biopsies.

In this proof-of-concept study, we demonstrated the feasibility of using FUS in combination with microbubbles for the local release of mRNA from glioblastoma tumors in mice into the bloodstream for liquid biopsies. We selected glioblastoma as our tumor model because it is the most frequent type of primary brain cancer in adults and associated with a dismal prognosis^27^. The biomarker we used was enhanced green fluorescent protein (eGFP) mRNA, which was highly specific to the tumor models used in this study, as the tumor models were established by the direct injection of eGFP-luciferase-transduced glioblastoma cells into the mouse brain. Human glioma U87 cells and murine glioma GL261 cells were used to develop two mouse models of glioblastoma.

## Results

### FUS-enabled liquid biopsy in an orthotopic human glioma xenograft model

**Figure 1:**
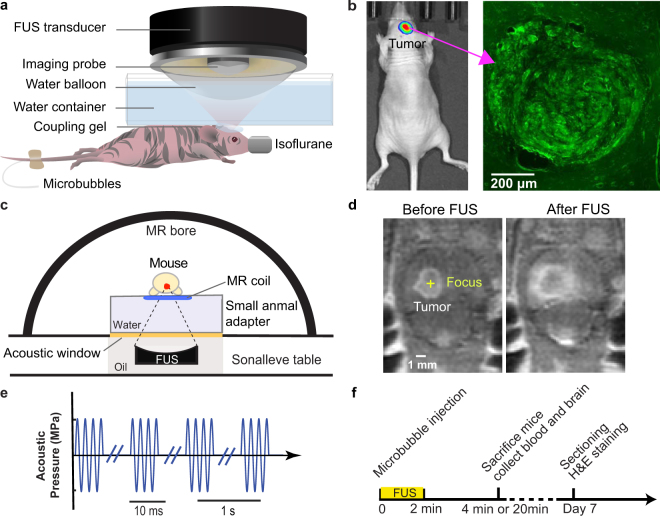
Experimental method. (**a**) Schematic illustration of the FUS experiment setup for the treatment of U87 tumor-bearing mice. (**b**) Bioluminescence image of the orthotopic mouse model with the green fluorescence image of the mouse brain shown on the right. (**c**) Schematic illustration of the MRgFUS system for the treatment of GL261 tumor-bearing mice. (**d**) Representative contrast-enhanced MR images acquired before and after FUS treatment. The enhanced accumulation of the MR contrast agents in the tumor region verified accurate tumor targeting by the FUS. (**e**) Diagram of FUS pulses. (**f**) Illustration of the experimental timeline.

An orthotopic mouse model developed by implantation of eGFP-luciferase-transduced human glioma cells (U87) into the brains of nude mice was treated by an ultrasound imaging-guided FUS system (Fig. 1a). Local growth of the tumors within the brain was assessed by monitoring luciferase activity using bioluminescent imaging (BLI) and verified using fluorescence imaging of the *ex vivo* brain slices (Fig. 1b). The focus of the FUS transducer (frequency = 1.5 MHz) was targeted at the tumor based on the tumor location identified by the BLI. The acoustic pressure of the FUS pulses was 3.82 MPa and other parameters were similar to those used for the BBB disruption (duty cycle of 1%, pulse repetition frequency of 1 Hz, and exposure duration of 2 min) (Fig. 1e)^20–24^. The experimental timeline is shown in Fig. 1f. For the U87 mice, terminal (non-survival) blood collection via cardiac puncture was performed immediately (~4 minutes) after FUS treatment. Such a short interval was selected in reference to a recent observation showing shorter collection time after FUS treatment was associated with higher RNA yield^13^.

**Figure 2:**
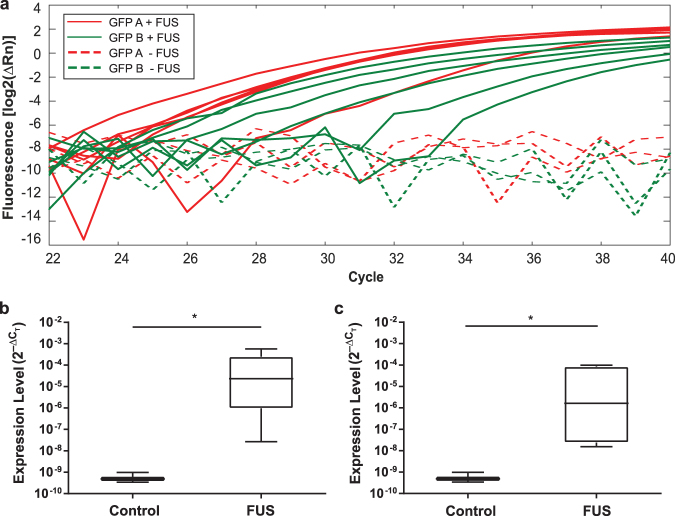
Comparison of the circulating eGFP mRNA expression in the control and treated U87 mice. (**a**) Amplification curves [log_2_(ΔRn)] of circulating eGFP mRNA in the control (n = 3) and treated mice (n = 6) for two primer pairs, eGFP A and eGFP B. ΔRn is the fluorescence intensity of eGFP mRNA minus the baseline. Comparison of the relative expression levels (${2}^{{-{\rm{\triangle }}{\rm{C}}}_{{\rm{T}}}}$) of (**b**) eGFP A and (**c**) eGFP B in the control and treated mice. *p < 0.05.

Quantitative polymerase chain reaction (qPCR) was performed to determine relative circulating levels of eGFP mRNA (target biomarker) normalized to 5.8S rRNA (internal control) in blood serum. Two pairs of PCR primers were used for the quantification of eGFP, namely eGFP A and eGFP B (Table 1). PCR products for the two eGFP primer pairs were undetectable in the control mice without FUS treatment with the qPCR maximum cycle number set to 40 (Table 2). Amplification curves of circulating eGFP mRNA in control and treated mice for the two primer pairs are shown in Fig. 2a. Quantification of the eGFP mRNA in blood collected from U87 mice found the average ± standard deviation of ΔC_T_ for eGFP A and eGFP B were both 30.7 ± 0.8 for the control group without FUS treatment. For the FUS-treated mice, the average ± standard deviation of ΔC_T_ for eGFP A and eGFP B were 16.6 ± 5.2 and 19.4 ± 5.4, respectively (Table 2).

**Table 1:** Forward and reverse primers used in qPCR for eGFP mRNA and 5.8S rRNA.

Primer	Forward	Reverse
eGFP A	AGAACGGCATCAAGGTGAAC	TGCTCAGGTAGTGGTTGTCG
eGFP B	TATATCATGGCCGACAAGCA	ACTGGGTGCTCAGGTAGTGG
5.8S rRNA	GACTCTTAGCGGTGGATCACT	CGTTCTTCATCGACGCACGA

Two primers were used for eGFP quantification, called eGFP A and eGFP B. 5.8S rRNA was used as an internal control.

**Table 2:** Summary of normalized cycle threshold, ${\rm{\Delta }}{C}_{T}$, for eGFP A and eGFP B in the U87 control mice (C1–C3; n = 3) and treated mice (T1–T6; n = 6).

Mice Identifier	eGFP A ${\boldsymbol{\Delta }}{{\boldsymbol{C}}}_{{\boldsymbol{T}}}$	eGFP B ${{\boldsymbol{\Delta }}{\boldsymbol{C}}}_{{\boldsymbol{T}}}$
**Control Mice**		
C1	29.9	29.9
C2	30.9	30.9
C3	31.4	31.4
**Treated Mice**		
T1	14.8	25.9
T2	19.6	19.3
T3	25.1	25.1
T4	12.8	13.6
T5	16.4	19.1
T6	10.8	13.3

${2}^{{-{\rm{\triangle }}{\rm{C}}}_{{\rm{T}}}}$ was calculated to compare the relative gene expression levels of the FUS-treated mice and the control mice (Fig. 2b,c). For both eGFP primer pairs, circulating mRNA levels of eGFP was significantly higher in the FUS-treated group compared with the untreated control group (eGFP A: *p* = 0.01; eGFP B: *p* = 0.01; one-tailed non-parametric Mann Whitney U Test).

### FUS-enabled liquid biopsy in an orthotopic murine xenograft glioma model

The second orthotopic glioma model was developed by direct implantation of murine glioma cells (GL261) into the Swiss mice. A magnetic resonance-guided FUS (MRgFUS) system was used for the FUS sonication to achieve accurate tumor targeting (Fig. 1c). The MRgFUS system, which was operated at 1.44 MHz, was targeted at the center of the tumor. Three groups of GL261 mice were treated by FUS with acoustic pressures of  1.52 MPa, 2.74 MPa, and 3.53 MPa, respectively. All other parameters were kept the same as those used in the treatment of U87 mice. Contrast-enhanced MR images were acquired before FUS sonication to identify the location of the tumor for FUS targeting and after FUS sonication to verify accurate tumor targeting by the FUS (Fig. 1d).

Quantification of the eGFP mRNA in the blood collected from GL261 mice found the average ± standard deviation of ΔC_T_ for eGFP A and eGFP B were both 23.6 ± 0.2 for the control group. The mean ± standard deviation of ΔC_T_ for eGFP A was 11.6 ± 0.1 for the 1.52 MPa group, 12.5 ± 0.4 for the 2.74 MPa group, and 12.2 ± 0.1 for the 3.53 MPa group (Table 3). The mean ± standard deviation of ΔC_T_ for eGFP B were 12.2 ± 0.3, 13.7 ± 0.7, and 13.0 ± 0.2 for the three groups, respectively (Table 3).

**Table 3:** Summary of normalized cycle threshold, ${\rm{\Delta }}{C}_{T}$, for eGFP A and eGFP B in the GL261 control mice (C1–C3; n = 3) and treated mice with different acoustic pressures.

Mice Identifier	eGFP A ${\boldsymbol{\Delta }}{{\boldsymbol{C}}}_{{\boldsymbol{T}}}$	eGFP B ${{\boldsymbol{\Delta }}{\boldsymbol{C}}}_{{\boldsymbol{T}}}$
**Control Mice**		
C1	23.6	23.6
C2	23.9	23.8
C3	23.4	23.4
**Treated Mice**		
1.52 MPa	11.6	12.4
1.52 MPa	11.5	11.8
1.52 MPa	11.7	12.3
2.74 MPa	12.5	13.9
2.74 MPa	12.0	12.8
2.74 MPa	12.9	14.5
3.53 MPa	12.3	13.2
3.53 MPa	12.2	13.1
3.53 MPa	12.1	12.8

**Figure 3:**
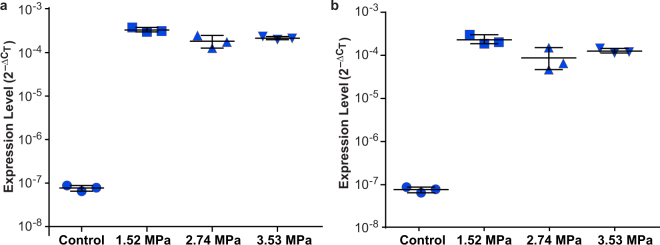
Comparison of the circulating eGFP mRNA expression in the control and treated GL261 mice. Comparison of the expression levels (${2}^{{-{\rm{\triangle }}{\rm{C}}}_{{\rm{T}}}}$) of (**a**) eGFP A and (**b**) eGFP B in the control group and three treatment groups with different acoustic pressures: 1.52 MPa, 2.74 MPa, and 3.53 MPa. All the measured data points as well as their mean and standard deviation are shown for each group. The circulating mRNA levels of eGFP A and eGFP B were significantly higher in the FUS-treated groups compared with the untreated control group (eGFP A: *p* = 0.0045; eGFP B: *p* = 0.0045). The expression levels of mice in the 1.52 MPa group was significantly higher than those of the other two groups for eGFP A and eGFP B (eGFP A: *p* = 0.012; eGFP B: *p* = 0.012).

Regardless of acoustic pressure, circulating eGFP levels were significantly higher (1,500–4,800 fold higher) in the FUS-treated groups (n = 9 in total, eGFP A: *p* = 0.0045; eGFP B: *p* = 0.0045; one-tailed Mann Whitney U Test) relative to the control group (n = 3, Fig. 3). The expression levels of mice treated at the lowest pressure (n = 3; 1.52 MPa) was significantly higher than those of the other two groups for eGFP A and eGFP B (n = 6; eGFP A: 1.7 fold increase in average, *p* = 0.012; eGFP B: 2.2 fold increase in average; *p* = 0.012; one-tailed Mann Whitney U Test). This finding suggests that the relatively lower pressure (1.48 MPa) was more efficient in releasing eGFP mRNA from the tumor than the relatively higher pressures (2.74 MPa and 3.53 MPa). Additionally, our preliminary MRI data found that the MR contrast enhancement ratios (calculated by the intensities of the MR images acquired after FUS treatment divided by the intensities of the images obtained before FUS treatment) were not significantly different among the three pressure groups (Supplementary Information).

### Histological examination

**Figure 4:**
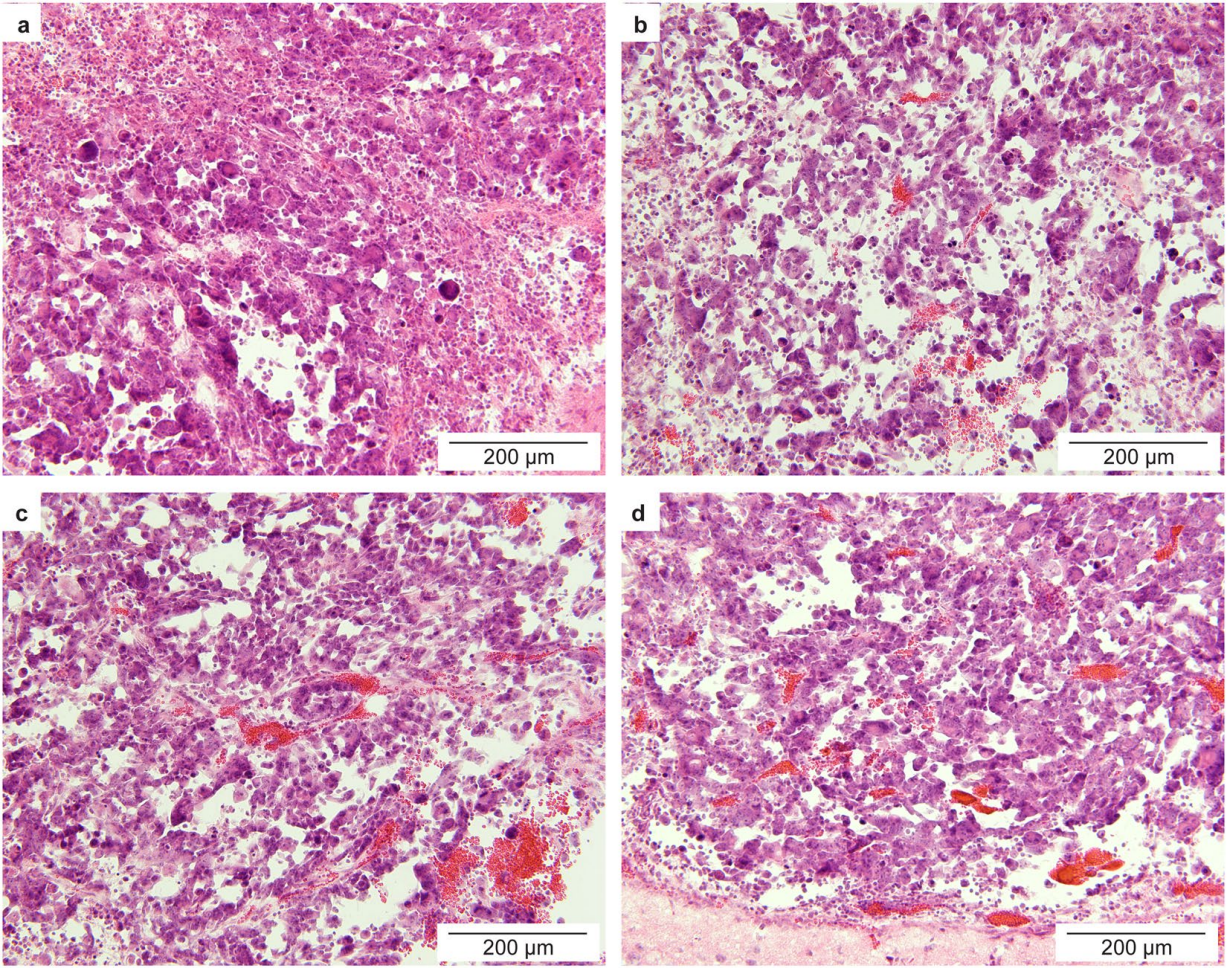
Histological assessment of brain tissue from the control and treated GL261 mice. H&E staining of the *ex vivo* tumor slices obtained from the control mice (**a**) and mice treated with FUS at (**b**) 1.52 MPa, (**c**) 2.74 MPa, and (**d**) 3.53 MPa, respectively. Hemorrhages were observed in all the FUS treated mice.

Hematoxylin and eosin (H&E) staining of the GL261 mouse brains found red blood cell extravasation in all mice treated with FUS (Fig. 4). More severe hemorrhage was observed in brain slices obtained from mice treated with the relatively higher pressures (2.74 MPa and 3.53 MPa) than mice treated with the relatively lower pressure (1.52 MPa). Vascular damage was expected as the acoustic pressures used in this proof-of-concept study were higher than the pressures normally used for the BBB opening without causing vascular damage. Of note, hemorrhage was not observed in the U87 mice treated by FUS at 3.82 MPa (Supplementary Information). The short interval between FUS sonication and animal scarification in the U87 mice (about 4 minutes vs. 20 minutes in GL261 mice) may have precluded the appearance of red blood cells in the brain slices even in the presence of tissue damage.

## Discussion

This study demonstrated the feasibility of FUS-enabled brain liquid biopsy using two glioma mouse models. FUS in combination with microbubbles achieved noninvasive and spatially-localized biomarker release from the brain tumor into the bloodstream. The noninvasive nature of the proposed FUS technique is especially advantageous over conventional neurosurgical tissue biopsies. Moreover, the technique presents a unique advantage in the assessment of spatially heterogeneous tumors. Tumor heterogeneity, which is a hallmark of glioblastoma^28^, poses a significant challenge to cancer biomarker research^29^. FUS can precisely target different locations of the tumor, thereby causing biomarkers to be released in a spatially-localized manner. By targeting multiple tumor regions in a single FUS session, the technique can be used to capture and analyze spatially heterogeneous biomarkers in a single liquid biopsy sample. Another potential functionality would be to perform multiple FUS sessions, each followed by a liquid biopsy, in order to detect specific biomarkers for each spatial location of the tumor to better understand the spatial heterogeneity of the tumor.

Both ultrasound-imaging guided FUS and MRgFUS treatment yielded substantial increase in eGFP RNA levels. However, the standard deviations of the qPCR measurement results for FUS-treated GL261 mice were lower than those of the FUS-treated U87 mice (Figs 2 and 3). This decrease in variations of the experimental results was associated with the use of the MRgFUS system, which improved the tumor targeting accuracy compared with the ultrasound imaging-guided FUS system.

Although damage was observed, the proposed FUS-enabled brain liquid biopsy technique has clear safety benefits compared over craniotomy for surgical biopsy of brain tissue. Moreover, the finding that among the three acoustic pressures the lowest pressure (1.48 MPa) achieved the highest eGFP mRNA expression level is important. It suggests that vascular damage associated with FUS treatment at the relatively higher acoustic pressures (2.74 MPa and 3.53 MPa) may hinder the efficient passage of tumor biomarkers into the peripheral circulation. It also suggests that successful biomarker release may be achievable with acoustic pressures lower than 1.48 MPa, which can improve the safety of the FUS technique. Relative higher acoustic pressures (1.52– 3.53 MPa) were used in the current study than that commonly used for the BBB disruption (*e*.*g*., 0.45 MPa), because in this proof-of-principle study we intended to enhance the interaction between microbubbles and the brain tissues. Future studies are needed to optimize the acoustic parameters (*e*.*g*., acoustic pressure, pulse repetition frequency, pulse length, and sonication duration) and microbubble parameters (*e*.*g*., size and dose) to explore the potential to achieve enhanced biomarker release with minimal or no tissue damage.

There are several limitations of this study. First, as mentioned earlier, the proposed technique needs to be optimized by assessing the efficiency of biomarker release under different FUS and microbubble parameters. Specifically, the feasibility to release biomarkers using FUS with lower pressures (*e*.*g*., 0.45 MPa) that are commonly used for BBB disruption without causing vascular damage needs to be evaluated. Second, the short-term and long-term safety of the FUS brain liquid biopsy technique need to be carefully assessed. Extracranial metastases of glioblastoma are rare^30^, and risk for tumor spread after surgical brain biopsies is considered negligible. We expect that the FUS liquid biopsies will not induce metastatic spread. Nevertheless, future studies are needed to determine the potential of metastasis associated with the FUS treatment. Third, the terminal cardiac puncture was used for blood collection as mice have a small total blood volume (~1.5 mL). Repeated blood sample collection was not feasible using the mouse model. Larger animal models (*e*.*g*., rats) are needed to collect blood samples at multiple time points after the FUS treatment. These blood samples can be used to assess the temporal dependency of the amount of biomarkers released by the FUS treatment and establish the optimal blood collection time. The larger animal models are also needed to evaluate the repeatability of this technique by performing the FUS treatment on the same animal in multiple days. Fourth, we quantified the contrast-enhanced MR images acquired before and after the FUS treatment (Fig. 1d). There was no significant difference in the MR contrast enhancement ratios among the three pressure groups (1.52 MPa, 2.74 MPa, and  3.53 MPa; Supplementary Information). This finding was not consistent with the quantification results of eGFR mRNA expression level shown in Fig. 3, which found the eGFR mRNA expression level in the 1.48 MPa group was significantly higher than that of the other two higher pressure groups. Future studies need to determine when lower acoustic pressures are used whether contrast-enhanced MRI is a useful tool in predicting the amount of biomarkers released by FUS. Fifth, we used an exogenous molecular marker (eGFP) to demonstrate the feasibility of FUS in noninvasive and spatially-focused liquid biopsy. Future studies are necessary to assess the generalizability of our findings to other tumor markers (*e*.*g*., DNA-based markers). Last but not least, the exact mechanism by which FUS-enabled release of molecular biomarkers is unknown. We propose that the mechanism may be that the FUS-induced BBB disruption opens a “two-way door,” allowing “two-way trafficking” between the brain and blood. Future studies will explore the potential mechanisms of FUS-enabled biomarker release.

## Conclusions

We demonstrated that the combination of FUS and microbubbles allows detection of tumor-specific eGFP mRNA in the bloodstream that is otherwise undetectable. Our findings established that FUS-mediated BBB disruption could enhance brain-to-blood trafficking. FUS may offer an enabling technique for noninvasive and regionally-specific brain tumor liquid biopsy that can be utilized in personalized brain cancer patient care.

## Materials and Methods

### Orthotopic mouse glioblastoma models

All animal procedures were reviewed and approved by the Institutional Animal Care and Use Committee of Washington University in St. Louis in accordance with the National Institutes of Health Guidelines for animal research.

Two orthotopic mouse glioblastoma models were developed: (i) NCl athymic NCr-nu/nu mice (Strain 553, Charles River Laboratory, Wilmington, MA, USA) injected with U87 human glioblastoma cells; and (ii) NIH Swiss mice (Strain 550, Charles River Laboratory, Wilmington, MA, USA) implanted with GL261 murine glioblastoma cells. Mice were anesthetized and fixed into a stereotactic head frame. A paramedian incision was made on the scalp, and a 1-mm burr hole was drilled 2 mm posterior and 1.5 mm lateral to the bregma. eGFP-Luciferase-transduced glioblastoma cells (U87 or GL261) were mixed with Corning^TM^ Matrigel (Catalog 356231, Corning Life Science, New York, USA) and injected through the burr hole using a syringe. The burr hole was sealed with bone wax, and the skin incision was glued together with tissue glue. The growth of the tumor was monitored using IVIS^®^ Spectrum *In Vivo* Imaging System (Model 124262, PerkinElmer, Ohio, USA) once every week for four weeks. At around fifth week after tumor cell implantation, mice were recruited in the study described below.

### Data availability statement

All relevant data are within the paper.

### Ultrasound imaging-guided FUS treatment of U87 mice

A total of nine mice with orthotopic U87 glioblastoma tumors were divided into two groups: treatment group (n = 6) and control group (n = 3). The treatment group was treated with FUS spatially targeted at the tumor site after intravenous injection of microbubbles. The control group received no FUS. For FUS treatment, mice were first anesthetized with 2% isoflurane and placed on a stereotactic frame. A FUS system (VIFU 2000; Alpinion US Inc., Bothell, WA, USA) sonicated the tumor using the following parameters: frequency = 1.5 MHz, peak negative pressure =3.82 MPa, pulse length = 10 ms, pulse repetition frequency = 1 HZ, duration = 30 s at each location, and 4 separate locations were treated for each tumor (Fig. 1a,e). The pressure amplitudes and beam dimensions of the FUS transducer were calibrated using a needle hydrophone (Onda, CA, USA) in a degassed water tank before the experiment. The pressures reported here were the peak negative pressures measured in water while accounting for 18% attenuation by the mouse skull^31^. The full width at half maximum (FWHM) of the axial beam and lateral beam were 6.04 mm and 0.62 mm, respectively. Before FUS sonication, microbubbles manufactured in house^32^ were injected into the mouse tail vein at a concentration of 8 × 10^8^ microbubbles per mL and a volume of 30 µL per mouse. The in-house manufactured microbubbles comprised of a 90 mol% 1,2-distearoyl-sn- glycero-3- phosphocholine (DSPC) and 10 mol% 1,2-distearoyl-sn-glycero-3-phosphoethanolamine-N-[methoxy(polyethylene gly-col)2000] (DSPE-PEG2000) (Avanti Polar Lipids, Alabaster, AL, USA) lipid-shell and a perfluorobutane (FluoroMed, Round Rock, TX, USA) gas-core. These microbubbles had a median diameter of 4–5 mm, which were isolated from a poly-dispersed microbubble distribution using a differential centrifugation method^31^.

### MRgFUS treatment of GL261 mice

A total of 12 mice with implantation of GL261 glioblastoma tumors in the brain were split into four groups: control group (n = 3) and three treatment groups (n = 3 for each group). The three treatment groups were treated using a clinical MRgFUS system (Sonalleve V2, Profound Medical Inc., Mississauga, Canada) equipped with a dedicated small animal adapter (FUS Instruments Inc., Toronto, Ontario, Canada) (Fig. 1c)^33^. The Sonalleve MRgFUS system included a 256-element phased array transducer mounted to a five-axis robot positioner and located inside a modified MRI patient table. The acoustic fields generated by the phased array transducer was calibrated by a fiber optic hydrophone using our previous published method^34^. The FWHM of the axial beam and lateral beam were 12.10 mm and 1.37 mm, respectively. The MRgFUS system was integrated into a clinical MRI scanner (Ingenia 1.5 T, Philips, Best, the Netherlands). The small animal adapter included a frame to hold an animal, a water reservoir, and a small animal MRI coil.

For the treatment of GL261 mice, mice were anesthetized with 1–2% isoflurane and placed on the small animal adapter. Optimark (gadoversetamide, a gadolinium-based contrast agent, 0.5 mmol/ml) was injected intravenously into the mice through the tail vein at a dose of 0.1 mmol/kg. Contrast-enhanced 3D, T_1_-weighted MRI images of the mouse brain were acquired for treatment planning, and the FUS targeted location was selected to be the center of the tumor (Fig. 1d). After intravenous injection of the same dose of microbubbles as that used for U87 mice, the GL261 mice were treated by the MRgFUS system using the following parameters: frequency = 1.44 MHz, pulse length = 10 ms, pulse repetition frequency = 1 Hz, and duration = 2 min. The three treatment groups were treated at different peak negative pressures: 1.52 MPa, 2.74 MPa, and  3.53 MPa, respectively. After treatment, contrast-enhanced MRI images were acquired for confirming successful tumor targeting by the FUS (Fig. 1d).

### Analysis of eGFP mRNA

Blood samples of 500–800 µL were collected from the heart about 4 min (U87) or 20 min (GL261) after the FUS treatment and prepared for qPCR analysis of eGFP mRNA. All the collected blood was centrifuged at 3,000 rpm for 10 minutes. The supernatant was collected. RNA samples were purified using the miRNeasy serum/plasma kit (Catalog no. 217184, Qiagen, USA). Agencourt RNAClean XP beads (Catalog no. A63987, Beckman Coulter Inc., USA) were used to further purify the RNA. The RNA was then reverse transcribed to cDNA using the Applied Biosystems^TM^ high-capacity cDNA reverse transcription kit (Catalog no. 4368814, Thermo Fisher Scientific, USA). Two eGFP primer pairs (Table 1) were designed using OligoPerfect^TM^ Designer (ThermoFisher Scientific, USA). 5.8S rRNA was used as an internal amplification control with its forward and reverse primer sequences also listed in Table 1. The quantitative real-time PCR was performed using SYBR^TM^ Green PCR master mix (Applied Biosystems^TM^). All of the PCRs were performed on a 7900HT Fast Real-Time PCR System (Catalog # 4329001, Thermo Fisher Scientific, USA) using the following protocol: the reaction mixture was heated at 95 °C for 10 min, followed by 40 cycles of 95 °C for 5 s and 60 °C for 30 s. Amplification and dissociation curves generated by the SDS2.3 (Applied Biosystems) software were used for gene expression analysis.

Duplicate reactions were run for each sample and each primer set. We used 5.8S rRNA as the internal control to normalize the PCR data by calculating cycle threshold change (ΔC_T_) by subtracting C_T_ of the eGFP (C_T,eGFP_) by the C_T_ of housekeeping gene, 5.8S rRNA (C_T,5.8S_). The gene expression level was determined using the ${2}^{{-{\rm{\triangle }}{\rm{C}}}_{{\rm{T}}}}$ method: ${2}^{-{\rm{\Delta }}{C}_{T}}={2}^{-({C}_{T,{eGFP}}-{C}_{T,5.8s})}.$ Maximum cycle number was set to 40. In order to assess the reliability of 5.8 S rRNA as the internal control, ${2}^{{-{\rm{C}}}_{{\rm{T}}}}$ was calculated and compared for the treated and control groups^35^. The ${2}^{{-{\rm{\triangle }}{\rm{C}}}_{{\rm{T}}}}$ of the control mice (1.8 × 10^−3^ ± 8.6 × 10^−4^) was not significantly different from the ${2}^{{-{\rm{\triangle }}{\rm{C}}}_{{\rm{T}}}}$ of the FUS-treated mice (1.1 × 10^−3^ ± 1.8 × 10^−3^; *p* = 0.38; two-tailed Mann-Whitney U test).

### Histological analysis

After blood collection, all the mice were transcardially perfused with 0.01 M phosphate-buffered saline and then with 4% paraformaldehyde, and their brains were harvested and prepared for paraffin sectioning. The mouse brains were horizontally sectioned to 15 μm slices and used for H&E staining.

### Statistical analysis

GraphPad Prism (v6.04, La Jolla, CA, USA) and R (v3.4.1, R Core Development Team, 2017) were used to perform statistical analyses. Given the non-normality of ${2}^{{-{\rm{\triangle }}{\rm{C}}}_{{\rm{T}}}}$ distribution (Shapiro-Wilk test <0.05), group comparisons were made using a non-parametric Mann Whitney U Test. A *p*-value < 0.05 was used to determine statistical significance.

## Electronic supplementary material

Supplementary information
