# Supplementary material for: Focused Ultrasound-enabled Brain Tumor Liquid Biopsy
Source: Sci Rep. 2018 Apr 26;8:6553. doi: 10.1038/s41598-018-24516-7 (PMC5919906; doi:10.1038/s41598-018-24516-7)
Supplement: Supplementary file 1 — Supplementary information [file 41598_2018_24516_MOESM1_ESM.pdf]

SUPPLEMENTARY INFORMATION

Focused Ultrasound-enabled Brain Tumor Liquid Biopsy

Lifei Zhu[1*], Galen Cheng[1*], Dezhuang Ye[2], Arash Nazeri[3], Yimei Yue[2], Weijun Liu[4], Xiaowei Wang[4], Gavin P. Dunn[5,6], Allegra A. Petti[7,8], Eric C. Leuthardt[1,2,5,9,10], Hong Chen[1,4 #]

1. Department of Biomedical Engineering, Washington University in St. Louis, Saint Louis, MO, 63130, USA.
2. Department of Mechanical Engineering and Materials Science, Washington University in St. Louis, Saint Louis, MO, 63130, USA.

3. Mallinckrodt Institute of Radiology, Washington University School of Medicine, Saint Louis, MO 63110, USA

4. Department of Radiation Oncology, Washington University School of Medicine, Saint Louis, MO, 63108, USA.

5. Department of Neurosurgery, Washington University School of Medicine, Saint Louis, MO 63110, USA

6. Center for Human Immunology and Immunotherapy Programs, Washington University School of Medicine, Saint Louis, MO 63110, USA

7. McDonnel Genome Institute, Washington University School of Medicine, Saint Louis, MO 63110, USA

8. Department of Medicine, Washington University School of Medicine, Saint Louis, MO 63110, USA

9. Department of Neuroscience, Washington University School of Medicine, Saint. Louis, MO 63110, USA

Contrast enhanced T1-weighted magnetic resonance (MR) images were acquire before and after FUS treatment of the GL261 mice using the MRgFUS system. The contrast enhancement due to MR contrast agent leakage was quantified for all the acquired images using a customized Matlab program. This program selected a non-sonicated region in each image to define the background intensity. Pixels that had intensities above the background were identified in each image. The intensities of these identified pixels were summed to define the contrast enhancement of each image. We then calculated the MR contrast enhancement ratios of images acquired at the same anatomic location before and after FUS treatment. The mean contrast enhancement ratio was calculated for each mouse by averaging the contrast enhancement ratios calculated for all images for each tumor. Figure S1 presents the quantified MR contrast enhancement ratios for all the three treatment groups with different pressure levels (1.52 MPa, 2.74 MPa, and 3.53 MPa). All the ratios were higher than one, confirming that FUS was targeted at the tumor and enhanced the tumor leakage of the MR contrast agents. However, there were no significant difference in the MR contrast enhancement ratio among the three pressure groups. This result was not consistent with the quantification of biomarker release shown in Fig. 3, which found the eGFR mRNA expression level in the 1.52 MPa group was significantly higher than that of the other two higher pressure groups. This finding suggests that at acoustic pressures that induce vascular damage, contrast-enhanced MRI, which quantifies the amount of MR contrast agent leakage from the blood to the brain tumor, cannot predict the amount of biomarkers released from the brain tumor to the blood. Future study needs to assess the feasibility to achieve FUS brain liquid biopsies using acoustic pressures that will not induce vascular damage and then evaluate the correlation between the leakage of MR contrast agents detected by contrast-enhanced MRI and biomarker release level in the blood.

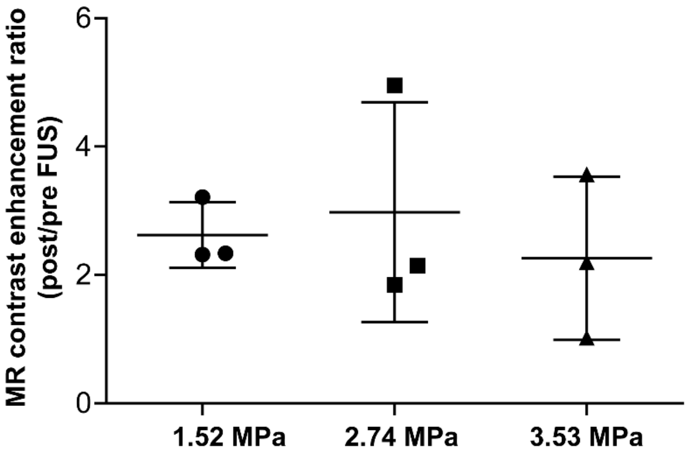

Figure S1. Quantification of contrast-enhanced MR images acquired before and after FUS treatment at the three pressure levels (1.52 MPa, 2.74 MPa, and 3.53 MPa). The lines represent the mean and standard deviation for each group. There was no statistically significant differences among all the groups.

Hematoxylin and eosin (H&E) staining was used to assess the effects of the focused ultrasound (FUS) treatment to the tumor tissue. The H&E staining of U87 tumor-bearing mice treated by FUS with acoustic pressure of 3.82 MPa did not reveal any red blood cells leakage in the brain tumor, which would have indicated hemorrhage (Figure S2). However, hemorrhages were observed in GL261 tumor-bearing mice treated with FUS with a similar pressure level (3.53 MPa) and even lower pressure levels (1.52 MPa and 2.74 MPa). The U87 tumor-bearing mice were sacrificed immediately (about 4 minutes) after the FUS treatment, which may have precluded the appearance of red blood cells in the brain slices even if any damage occurred.

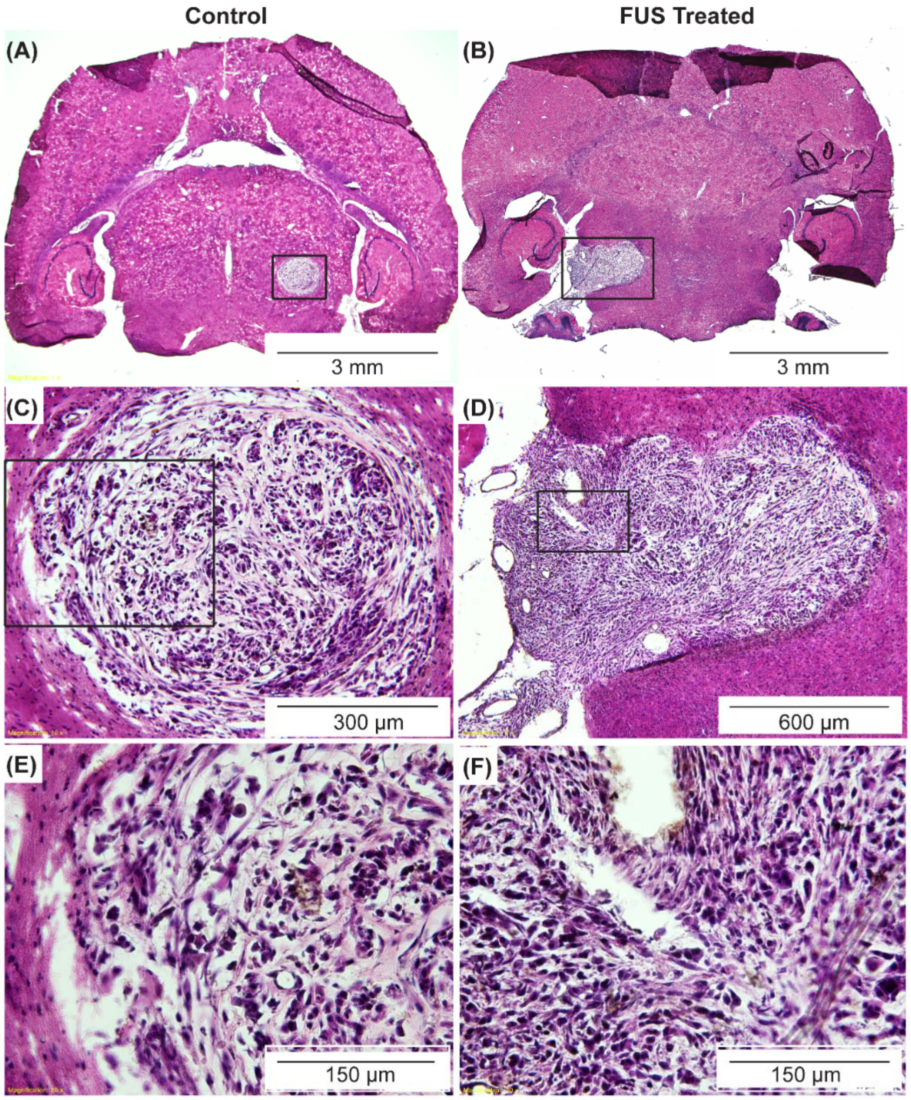

Figure S2. Histological assessment of brain tissue from the control and treated mice. *H&E staining of the ex vivo tumor slices obtained from the control and treated mice showing no acute damage in FUS-treated tumor (B, D, and F) when compared with the control (A, C, and E). The black boxes in A and B indicate the locations where C and E were obtained, respectively. The black boxes in C and E indicate the locations where E and F were obtained, respectively.*
